# Epidemiology and genetic evolution of dengue viruses in the French Pacific Territories

**Published:** 2011-01-10

**Authors:** Maite Aubry, Myrielle Dupont-Rouzeyrol, Olivia O’Connor, Claudine Roche, Stéphane Lastère, Suzanne Chanteau, John Aaskov, Van-Mai Cao-Lormeau

**Affiliations:** 1 Institut Louis Malardé, Papeete, 98713, French Polynesia; 2 Institut Pasteur de Nouvelle-Calédonie, Nouméa, 98800, New Caledonia; 3 WHO Collaborating Centre for Arbovirus Reference and Research, Queensland University of Technology, Brisbane, Queensland 4001, Australia

## 

During the last decades, whole chains of dengue epidemics emerged in the South Pacific region. In contrast with the situation in hyper endemic continental countries and in the Caribbean, the epidemiology of dengue in the South Pacific islands Countries (SPICs) is characterized by the non-persistent co-circulation of multiple serotypes and the long-term predominance, with local re-emergences, of a single genotype. Local specificities in the epidemiological profile of dengue can also been observed between the SPICs, probably related to differences in the geographical situation, the eco-biological context (climate, endemic mosquito species), the demography and the population flew. In the present study, by focusing on the past DEN3_1989-96_ and the recent DEN1_2001-10_ and DEN4_2007-10_ circulation periods, we addressed the question of the circulation of dengue viruses between the French Pacific Territories and the impact of the “local context” on viral genetic evolution. Hundreds of viral strains collected during both epidemic and endemic periods in French Polynesia (FP), New Caledonia (NC) and Wallis & Futuna (WF) were sequenced on the complete E gene. The phylogenetic analysis corroborates the previous observations on the predominant circulation of a single genotype. Within each serotype/genotype, the viral strains collected in the SPICs formed a “Pacific clade”. Within this clade, the strains from the French Pacific Territories formed a unique lineage during the early epidemic/endemic circulation periods but diverged in distinct lineages when the virus re-emerged. By analyzing the in time/in space fixations of genetic mutations on the E gene, we observed that some mutations are shared by the French Pacific Territories but differ from the other SPICs. Moreover, although the majority of the mutations acquired in FP are also found on NC and WF strains, some seem to be specific to the Territory. Our results support the hypothesis of an impact of both the regional and local contexts on the genetic evolution of dengue viruses.

